# Epidemiological Surveillance of Lymphocryptovirus Infection in Wild Bonobos

**DOI:** 10.3389/fmicb.2016.01262

**Published:** 2016-08-12

**Authors:** Tomoyuki Yoshida, Hiroyuki Takemoto, Tetsuya Sakamaki, Nahoko Tokuyama, John Hart, Terese Hart, Jef Dupain, Amy Cobden, Mbangi Mulavwa, Yoshi Kawamoto, Akihisa Kaneko, Yuki Enomoto, Eiji Sato, Takanori Kooriyama, Takako Miyabe-Nishiwaki, Juri Suzuki, Akatsuki Saito, Munehiro Okamoto, Masaki Tomonaga, Tetsuro Matsuzawa, Takeshi Furuichi, Hirofumi Akari

**Affiliations:** ^1^Primate Research Institute, Kyoto UniversityKyoto, Japan; ^2^Lukuru Wildlife Research FoundationKinshasa, Congo; ^3^African Wildlife FoundationNairobi, Kenya; ^4^Department of Anthropology, Emory University, AtlantaGA, USA; ^5^Research Center for Ecology and Forestry, Ministry of High Education and Scientific ResearchMabali, Congo; ^6^Department of Veterinary Science, School of Veterinary Medicine, Rakuno Gakuen UniversityHokkaido, Japan; ^7^Institute for Virus Research, Kyoto UniversityKyoto, Japan

**Keywords:** bonobo, lymphocryptovirus, epidemiology, surveillance, feces, apes

## Abstract

Lymphocryptovirus (LCV) is one of the major gena in the herpesvirus family and is widely disseminated among primates. LCVs of human and rhesus macaques are shown to be causative agents of a number of malignant diseases including lymphoma and carcinoma. Bonobos (*Pan paniscus*) are highly endangered and the least studied species of the great apes. Considering the potential pathogenicity of the LCV that might threaten the fate of wild bonobos, population-based epidemiological information in terms of LCV prevalence in different location of Bonobo’s habitats will help propose improved conservation strategies for the bonobos. However, such data are not available yet because it is very difficult to collect blood samples in the wild and thus virtually impossible to conduct sero-epidemiological study on the wild ape. In order to overcome this issue, we focused on evaluating anti-LCV IgA in the feces of bonobos, which are available in a non-invasive manner. Preliminary study showed that anti-LCV IgA but not IgG was efficiently and reproducibly detected in the feces of captive chimpanzees. It is noteworthy that the fecal IgA-positive individuals were seropositive for both anti-LCV IgG and IgA and that the IgA antibodies in both sera and feces were also detectable by Western blotting assay. These results indicate that the detection of fecal anti-LCV IgA is likely a reliable and feasible for epidemiological surveillance of LCV prevalence in the great apes. We then applied this method and found that 31% of wild bonobos tested were positive for anti-LCV IgA antibody in the feces. Notably, the positivity rates varied extensively among their sampled populations. In conclusion, our results in this study demonstrate that LCV is highly disseminated among wild bonobos while the prevalence is remarkably diverse in their population-dependent manner.

## Introduction

Genetic studies have shown that chimpanzees and bonobos diverged within the last million years or so (Becquet and Przeworski, 2007; Hey, 2010; Takemoto et al., 2015). Chimpanzees remain widespread in tropical Africa, occurring in a variety of habitats, ranging from tropical forests to savannah mosaics. Estimates of total surviving numbers have increased from about 200,000 in the 1980s to a maximum of approximately 300,000 in 2003 (Oates, 2006). This growth in population is due in large part to conservation efforts by humans. By contrast, bonobos inhabit only Democratic Republic of the Congo (DRC); they are the least studied species of the great apes, and efforts to protect them and their habitats were significantly hindered as a result of civil war spanning the late 1990s and early 2000s (Vogel, 2000). Prior to the civil war, their population was estimated at around 29,500–50,000^1^. Their numbers have likely been reduced to a half of pre-war estimates as a result of habitat loss and concurrent increases in hunting over the last two decades (Nackoney et al., 2014). Due to limitations in both research and conservation in bonobo habitats, we still understand little in terms of what ecological factors (disease, habitat fragmentation, hunting, resource distribution, etc.) play the most important roles in their health and mortality. It is critical to continue ecological studies regarding endangered wild bonobos in order to better understand what factors are most important in their survival (Hickey et al., 2013).

In the last decade, the connection between African great apes and emerging diseases such as AIDS, malaria, and Ebola has gained considerable attention, as it relates to global health and great ape conservation (Ryan and Walsh, 2011). Wild populations of gorillas and chimpanzees are threatened by a diverse array of virulent pathogens, including Ebola virus (Walsh et al., 2003; Bermejo et al., 2006; Le Gouar et al., 2009), simian immunodeficiency virus (SIV; Keele et al., 2006, 2009), in addition to a variety of human respiratory viruses (Skiadopoulos et al., 2002; Kaur et al., 2008; Kondgen et al., 2008; Szentiks et al., 2009). Bermejo et al. (2006) estimated a loss of approximately 5000 gorillas in West Africa due to endemic Ebola outbreaks in 2002/2003. SIV isolated from chimpanzees (SIVcpz) is now known to be pathogenic in chimpanzees (Keele et al., 2009).

Lymphocryptovirus (LCV) belongs to the genus gammaherpesvirus. Much like SIV and malaria, LCV usually exhibits latent infection, where stress and/or immune suppression can cause both viral expression and subsequent onset of a variety of diseases (Mühe and Wang, 2015). LCV is widely disseminated among primates, including humans and chimpanzees (Mühe and Wang, 2015). Epstein–Barr virus (EBV) is a widely recognized human-specific LCV and is a causative agent of infectious mononucleosis, Hodgkin’s lymphoma, Burkitt’s lymphoma, and nasopharyngeal carcinoma (Macsween and Crawford, 2003). LCV is naturally infected with great apes including chimpanzees and gorillas and is closely related with EBV in terms of genomic organization and biological properties (Rivailler et al., 2002a,b). It is therefore reasonable to assume that the LCV may also be pathogenic to the great apes as natural hosts. So far epidemiological information regarding LCV prevalence in wild bonobos is not available. Considering the potential pathogenicity of the LCV that might threaten the fate of wild bonobos, in the present study we aimed at examining the prevalence of LCV in wild bonobos, which may help propose improved conservation strategies for the bonobos. However, it was not possible to perform usual serological surveillance since sera samples of the bonobos were unavailable. A previous report showed that some of EBV-infected humans became positive for anti-EBV IgA (Hanlon et al., 2014), which led us to come up with an idea that anti-LCV IgA could be detectable in the feces. In order to test the possibility, we needed to overcome the following hurdles; (i) if the great apes infected with LCV could become positive for IgA as well as IgG against LCV in sera similar to human cases, (ii) if the anti-LCV IgA level could be correlated with that of IgG in sera, considering the possibility that individuals infected with LCV were negative for anti-LCV IgA while were positive for anti-LCV IgG, and (iii) if the IgA and/or IgG could be efficiently eluted and detectable in the feces. In our recent pilot study of serological surveillance toward zoonotic and anthropozoonotic agents in captive chimpanzees at Primate Research Institute, Kyoto University (KUPRI), most of the chimpanzees were seropositive for LCV (Kooriyama et al., 2013). Taking these issues into consideration, we sought to address the above questions.

## Materials and Methods

### Animals

This study was conducted under the guidelines provided by KUPRI after the approval of Animal Welfare and Care Committee in Kyoto University. The chimpanzees employed in this study were kept in indoor-outdoor enclosure in KUPRI (Matsuzawa, 2003, 2006). Individual information of each chimpanzee is described in our previous report (Kooriyama et al., 2013). Chimpanzees were used for behavioral, psychological, and evolutional study and their health condition was monitored daily and also by a periodical health examination. None of these chimpanzees were previously vaccinated against any pathogens.

We observed bonobos (*Pan paniscus*) in Wamba, Tshuapa-Lomami-Lualaba landscape (TL2), Lomako, and Lake Tumba in Congo (Kawamoto et al., 2013). Research permission: MIN.RS/SG/003/2010 (Wamba), 024/ICCN/BP-MA/2010 (TL2), 008//MINRS/CREF/MAB/DG/01MNIK/2011 (Lac Tumba), 051/ICCN/DG/ADG/KV/2011 (Lomako).

### Sample Collection

Blood samples from captive chimpanzees were collected when each chimpanzee was anesthetized for periodical health examinations between 2007 and 2012. The chimpanzees were anesthetized with a combination of ketamine hydrochloride (Ketalar, Sankyo-Parke-Davis & Co., Inc., Japan, 3.5 mg/kg) and medetomidine hydrochloride (Domitor Meiji Seika Kaisha, Ltd, Tokyo, Japan, 0.035 mg/kg) with or without premedication with oral midazolam (1 mg/kg) or droperidol (0.2 mg/kg). Anesthesia was maintained with isoflurane (Isoflu, Dainippon Sumitomo Pharma, Co., Ltd, Osaka, Japan) or sevoflurane (Sevoflo, Dainippon Sumitomo Pharma, Co., Ltd, Osaka, Japan) when necessary. Sera were separated from the blood by centrifugation at 3,000 *g* for 20 min, and then analyzed within a day or stored at -80°C. Fecal samples from these chimpanzees were also collected into 50 ml tubes, and then stored at -80°C until extraction and purification.

Fecal Samples of wild bonobos were collected from four distinct populations in DRC. Wamba population is situated the north central of Congo basin in Luo Scientific Reserve (Furuichi et al., 2012). The three study groups, E1, PE, and PW, ranged from mainly primary or old secondary rainforest and swamp forest but also included young secondary forest and cultivated fields near the villages (Hashimoto et al., 1997; Terada et al., 2015). All members of the three groups were identified (Furuichi et al., 2012; Sakamaki et al., 2015). The Iyemba study site, located within the greater Lomako Reserve, is located at about 200 km to the west of the Wamba area. The Iyemba study site is mainly composed of undisturbed primary forest with sub-canopy dominated by the presence of *Marantacea* species (e.g., *Haumania liebrechtsiana*). Small streams run through the site, but swamp and seasonally inundated forest are uncommon, in contrast to other study sites with more heterogeneity and forest mosaic compositions, such as Wamba and Lui Kotal (Dupain et al., 2000; Hohmann and Fruth, 2003; Terada et al., 2015). We collected samples from two neighboring groups in Iyemba and Tolinda sites that are approximately 9 km apart. The Lac Tumba population is located near the Western limits of bonobo habitat in Congo basin (Inogwabini et al., 2008). The Bokonzoli and Botuwali sites, south of Lake Tumba, were surveyed. TL2 lies on the eastern limits of bonobo range^2^. Fecal Samples of wild bonobos were collected from regions around Katopa, Oluo, and Obenge campsites and near Bafundo village. The southern area of TL2 is occupied with savanna-forest mosaic vegetation.

Our methods for collection of bonobo feces varied according to the degree of their habituation. Since feces decay and disappear quickly, normally less than a half day after defecation in tropical forests, we collected fresh samples during our direct observation and placed into 50 ml tubes containing silica gel (Wako). For well-habituated communities, such as the Wamba E1 group, parties were followed from nest to nest everyday and feces were collected immediately following observation of defecation. During detailed behavioral follows, as with the Wamba E1 group, the names of individuals were recorded for each sample; we collected samples from each individual of the E1 group over a 2 months long survey. The Iyemba group was semi-habituated at the time of collection and individual identification was not always possible. Fecal samples were collected fresh from beneath night nests that had been located the previous evening. Samples were collected in this manner four times at Iyemba, and once in Tolinda during 1-month survey. In the other two sites, Lac Tumba and TL2, fecal samples were hard to find because no research had previously been conducted there. We gathered information on bonobos from villagers or local hunters and explored in the forest with local staff moving our camp for several weeks. We found several old nest sites, and samples were successfully collected once under the fresh nest groups at each site in Lac Tumba. A few samples were collected from nest groups in TL2. Encounter and near-miss with bonobos happened at Katopa, Oluo, and Bafundo. We could track their footprints to get fresh fecal samples. When we found feces under nests, we estimated the age of the nest (most of them were within a day), the number of nests and the latitude and longitude using GPS. We also estimated the size (large, medium, small) and hardness (solid, intermediate, soft: diarrhea-like) to check the general health condition. In the case of Wamba E1 group, we checked health status, such as sneezing or nasal mucus, for each individual everyday. Each thumb-sized sample was put into silica gel and alcohol tube using twigs from fallen branches.

### Serological Surveillance for LCV Infection in Chimpanzees

Serological surveillance for LCV infection in chimpanzees was conducted using an enzyme immunoassay (EIA) for detecting anti-EBV IgG since it was shown to be cross-reacted with the chimpanzee LCV (Kooriyama et al., 2013). EIA analysis was outsourced to Tokai Chuo Laboratory (ISO15189: 2003) in FALCO Biosystems, Ltd, Japan (Kooriyama et al., 2013).

### Extraction and Purification of IgA From Fecal Samples

Extraction of IgA from fecal samples was performed as previously described (Okhuysen et al., 1995; Santiago et al., 2003) with modifications. Fecal samples (∼10 g) were resuspended (10% [wt/vol]) in T-PER Tissue Protein Extraction Reagent (Thermo SCIENTIFIC) containing 1% aprotinin (SIGMA) and 1% protease inhibitor cocktail (SIGMA), vortexed, and centrifuged twice at 3,000 rpm (1,600 *g*) for 15 min at 4°C and at 6,000 rpm (3,300 *g*) for 30 min at 4°C to extensively remove solid matter. The supernatants were added with 1% aprotinin (SIGMA) and 1% protease inhibitor cocktail (SIGMA) and were stored at -80°C until use.

Purification of Igs from the supernatants was performed by NAb protein A/G Spin Kit (Thermo SCIENTIFIC) according to the manufacturer’s instruction. Column in a collection tube was centrifuged at 5,000 *g* for 1 min at room temperature (RT) to remove the storage solution. Four hundred μl of a binding buffer was added to the column/collection tubes to equilibrate the column. The column/collection tubes were centrifuged at 5,000 *g* for 1 min at RT to remove the binding buffer. Five hundred μl of antibody-containing fecal samples were added to the column, incubated at RT for 10 min. The column in the new collection tube was centrifuged at 5,000 *g* for 1 min at RT to remove the unbound sample components. The columns were washed with the binding buffer three times. An elution buffer was added to the columns/collection tube containing a neutralizing buffer, centrifuged at 5,000 *g* for 1 min at RT. Buffer was exchanged by Zeba desalt spin columns. The antibody-containing samples in the storage buffer were stored at 4°C until use.

### Detection of Antibodies Taken from Sera and Feces against LCV by Enzyme-Linked Immunosorbent Assay (ELISA)

The anti-LCV IgA antibodies from sera and feces were evaluated by using EBV the viral capsid antigen (VCA) IgA ELISA Kit (IBL INTERNATIONAL). The assay was performed according to the manufacturer’s instruction. To detect the anti-LCV VCA IgA in the samples from chimpanzees and bonobos, they were diluted in a dilution buffer, applied to the wells in serial dilutions, incubated for 1 h at 37°C and washed with the wash buffer three times. Anti-human IgA conjugated to horseradish peroxidase (HRP) was added to each well and incubated for 1 h at 37°C. Each well was washed with a wash buffer three times. Tetramethylbenzidine (TMB) substrate solution was added to each well and incubated for 30 min RT, and then the reaction was stopped with TMB stop solution. Optical density was measured using an enzyme-linked immunosorbent assay (ELISA) reader at 450 nm. Concentration of an anti-EBV IgG antibody from fecal samples was assessed by using the EBV VCA IgA ELISA kit with slight modifications. An EBV VCA antibody (abcam) was used as a standard and an HRP-human IgG Fab antibody (BETHYL) was used as a secondary antibody.

Concentration of an anti-EBV early antigen (EA) IgA antibody from sera and fecal samples was assessed by using ELISA construction system (IMMUNOtek) and EBV EA protein (abcam). The assay was performed according to the manufacturer’s instruction with a slight modification. The EBV EA antigen was dissolved in binding buffer (ZeptoBind). To detect the anti-EA IgA in sera and fecal samples, 96-well ELISA plates were coated with an EBV EA antigen and were incubated for overnight at 4°C. The plates were treated with blocking solution (ZeptoBlock) for 2 h at RT, then with coating buffer (ZeptoCaot) for 1 h at RT and dried for overnight at RT. The plates and a desiccant pillow were stored in a resealable bag at 4°C until use. To detect the anti-EBV EA IgA, the samples were diluted in a dilution buffer, applied to the wells in serial dilutions, incubated for 1 h at 37°C and washed with the wash buffer three times. The anti-human IgA conjugated with HRP was added to each well and incubated for 1 h at 37°C. Each well was washed with a wash buffer three times. TMB substrate solution was added to each well and incubated for 30 min at RT, and then the reaction was stopped with TMB stop solution. Optical density was measured with an ELISA reader at 450 nm.

### Detection of Fecal Antibodies against LCV by Western Blotting (WB) Analysis

The anti-LCV IgA from plasma or fecal samples was assessed by using Can Get Signal system (TOYOBO). Enhanced chemiluminescent was performed according to the manufacturer’s instructions and as previously described (Santiago et al., 2002, 2003) with slight modifications. WB strips transferred with total EBV proteins (Abcam) were blocked with 5% bovine serum albumin (SIGMA) in PBS-Tween (PBS-T) for 1 h at RT, and washed with the PBS-T three times. The blot strips were incubated with sera or purified fecal extracts in Can Get Signal solution 1 for 1 h at RT, and washed with the PBS-T three times. The blot strips were reacted with the goat anti-human IgG+IgM+IgA (1:200,000) conjugated to HRP (BETHYL) for 1 h at RT, and washed with PBS-T three times. The strips were examined for chemiluminescence by using an ECL Western Blotting Detection system (Amersham). A monoclonal antibody against EBV EA (Abcam) was used as a positive control (PC) for the assay. The EBV-negative human serum and the fecal elute obtained from a chimpanzee negative for the LCV EA antibody were employed as a negative control (NC), respectively.

## Results

### Comparison between Anti-LCV IgG and IgA Antibodies in Sera and Fecal Samples of Captive Chimpanzees

The purpose of this study was to survey prevalence of LCV infection in wild bonobos. However, it was not possible to perform usual serological surveillance since sera samples of the bonobos were unavailable. A previous report showed that some peoples infected with EBV were positive for anti-EBV IgA (Hanlon et al., 2014), which led us to come up with an idea that anti-LCV IgA could be detected in the feces elutes. In order to test the possibility, we needed to overcome the following hurdles; (i) if the great apes infected with LCV could become positive for IgA as well as IgG against LCV in sera similar to human cases, (ii) if the anti-LCV IgA level could be correlated with that of IgG, considering the possibility that individuals infected with LCV were negative for anti-LCV IgA while positive for anti-LCV IgG, and (iii) if the IgA and/or IgG could be efficiently eluted and detectable in the feces. We initially sought to address these questions.

**FIGURE 1:**
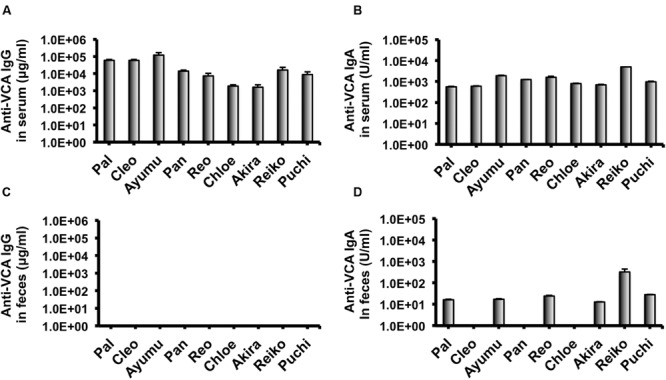
**Detection of IgG and IgA antibodies against LCV VCA in sera and feces of captive chimpanzees.** Antibodies against LCV VCA in sera **(A,B)** and feces **(C,D)** of captive chimpanzees were evaluated by ELISA. Results shown are representative of three independent experiments. The results of anti-VCA IgG **(A,C)** and the anti-VCA IgA **(B,D)** are indicated, respectively.

**FIGURE 2:**
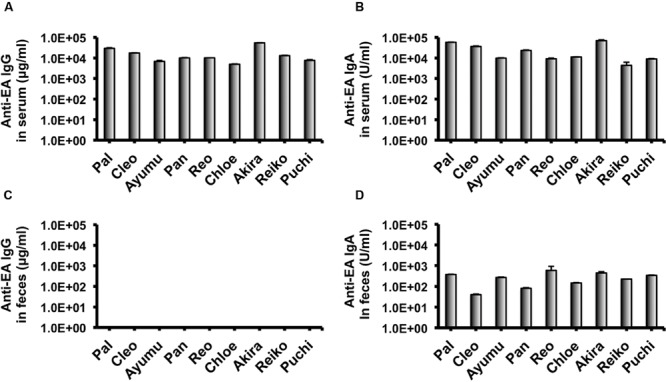
**Detection of IgG and IgA antibodies against LCV EA in sera and feces of captive chimpanzees.** Antibodies against LCV EA in sera **(A,B)** and feces **(C,D)** of captive chimpanzees were evaluated by ELISA. Results shown are representative of three independent experiments. The results of anti-EA IgG **(A,C)** and the anti-EA IgA **(B,D)** are indicated, respectively.

**FIGURE 3:**
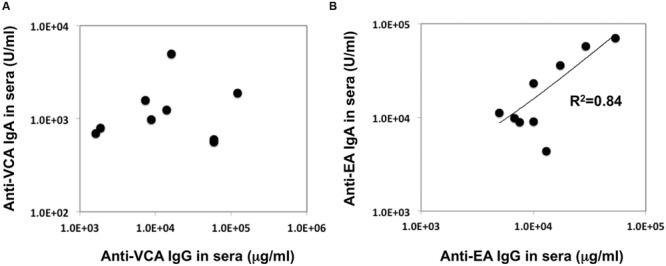
**Relationship between the concentration of the anti-VCA and EA IgA and IgG antibodies in the sera of captive chimpanzees.** The data regarding the concentration of IgA and IgG antibodies against LCV VCA **(A)** and EA **(B)** in the sera of captive chimpanzees as measured by ELISA were plotted.

**FIGURE 4:**
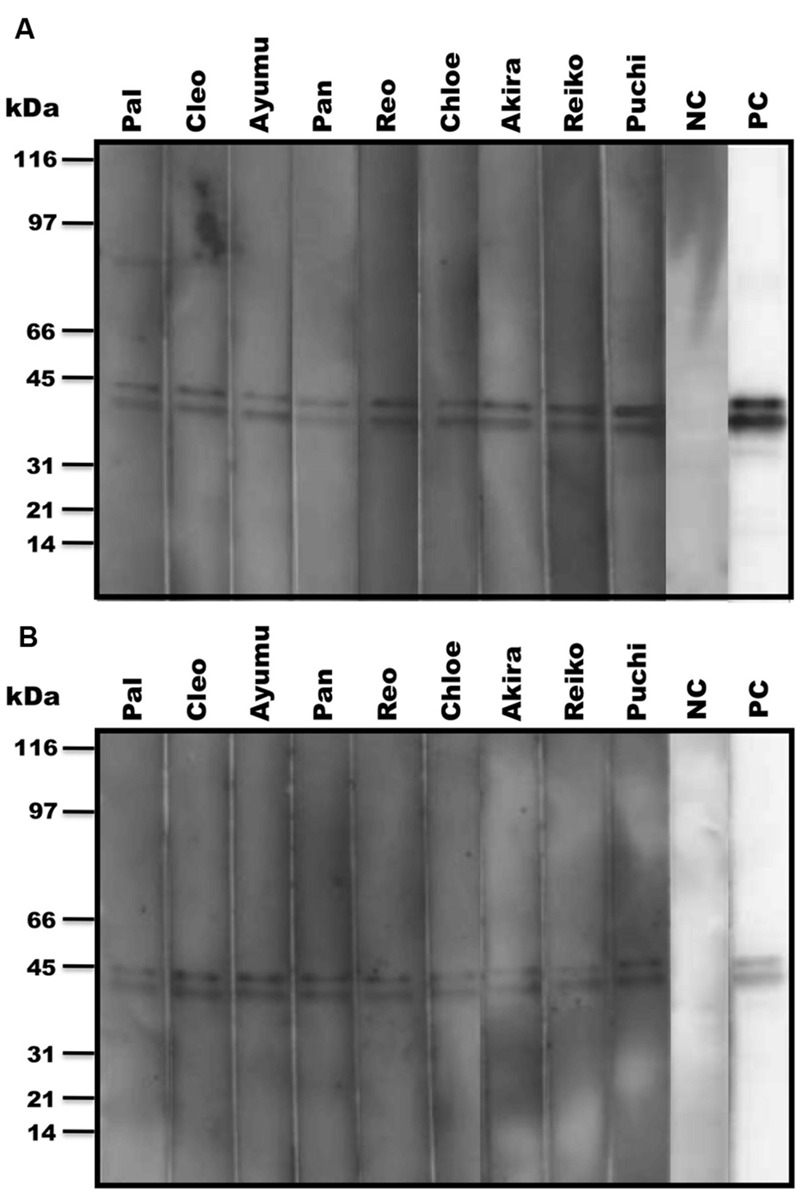
**Detection of LCV EA-specific antibodies in sera and fecal samples from captive chimpanzees by WB analysis.** The anti-LCV EA antibodies in sera **(A)** and feces **(B)** of captive chimpanzees were examined for the reactivity to EBV EA by WB analysis. PC and NC indicate positive and negative controls, respectively.

In our recent pilot study of serological surveillance toward zoonotic and anthropozoonotic agents in captive chimpanzees at KUPRI, most of our captive chimpanzees were seropositive for LCV (Kooriyama et al., 2013). In order to ask if the great apes infected with LCV could become positive for IgA as well as IgG against LCV in sera similar to human cases, the sera from the chimpanzees positive for anti-LCV IgG were evaluated for the anti-LCV IgA. EBV infection in humans is usually determined by detecting antibodies to VCA (van Grunsven et al., 1993). Moreover, it was reported that antibodies reactive to EBV EA as well as VCA was detected in apes such as orangutans, gorillas, and chimpanzees (Ishida and Yamamoto, 1987). Thus, we tested both the antigens in order to evaluate LCV-specific antibodies in the sera. We found that VCA-specific IgA antibody was detected in the sera of the chimpanzees positive for anti-VCA IgG (**Figures 1A,B**) and that VCA-specific IgA but not IgG was detected in the feces of six of nine individuals (**Figures 1C,D**). Similarly, EA-specific IgA as well as IgG antibodies were observed in the sera of these chimpanzees while the amounts of the EA-specific IgA were much greater than those of VCA-specific IgA (**Figures 2A,B**). In addition, the amounts of the EA-specific but not anti-VCA IgA and IgG among the individuals were significantly correlated (**Figure 3**). Importantly, the chimpanzees exhibited greater and detectable EA-specific IgA but not IgG antibody in their feces as compared with the case of the anti-VCA IgA (**Figures 2C,D**). These results indicated that the LCV-infected chimpanzees became seropositive for IgA as well as IgG against LCV, and also that the LCV EA-specific IgA but not IgG was efficiently detected in the feces. In order to confirm the reliability of the anti-EA IgA in the sera and the feces as detected by the ELISA assay, the samples were also subjected for WB analysis. We observed EA-specific bands in both the sera and the feces samples from the individuals positive for the anti-LCV EA IgA by the ELISA assay (**Figure 4**). Taken together, we concluded that the detection of fecal anti-LCV IgA was likely reliable and feasible for epidemiological surveillance of LCV prevalence in the great apes.

### Epidemiological Surveillance of LCV in Fecal Samples of Wild Bonobos

**FIGURE 5:**
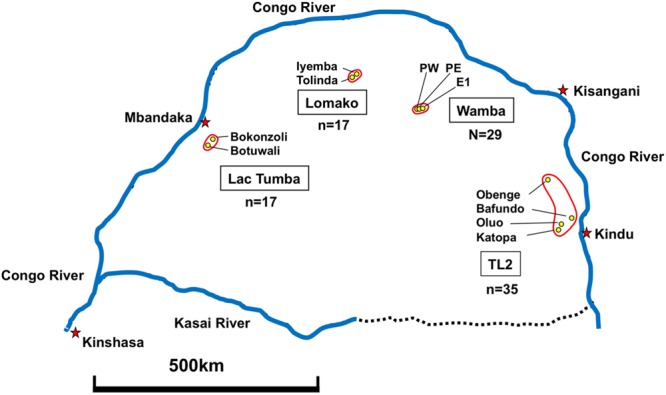
**Geographical map showing fecal sample collection sites in DRC.** Collection of 98 feces was performed as described in Section “Materials and Methods.”

We examined 98 fecal samples from wild bonobos across multiple field sites in the DRC for the prevalence of LCV infection. **Figure 5** shows sample numbers and locations where individual feces were collected. In order to recognize bonobo feces individually we performed microsatellite marker analysis by using DNA obtained from fecal samples (Kawamoto et al., 2013). Since only two fecal samples were derived from the same individual, we deleted one of them for further epidemiological analysis of LCV infection. As a result, 29 fecal samples from Wamba, 35 from TL2, 17 from Lomako, and 17 from Lac Tumba were employed for further analyses.

**FIGURE 6:**
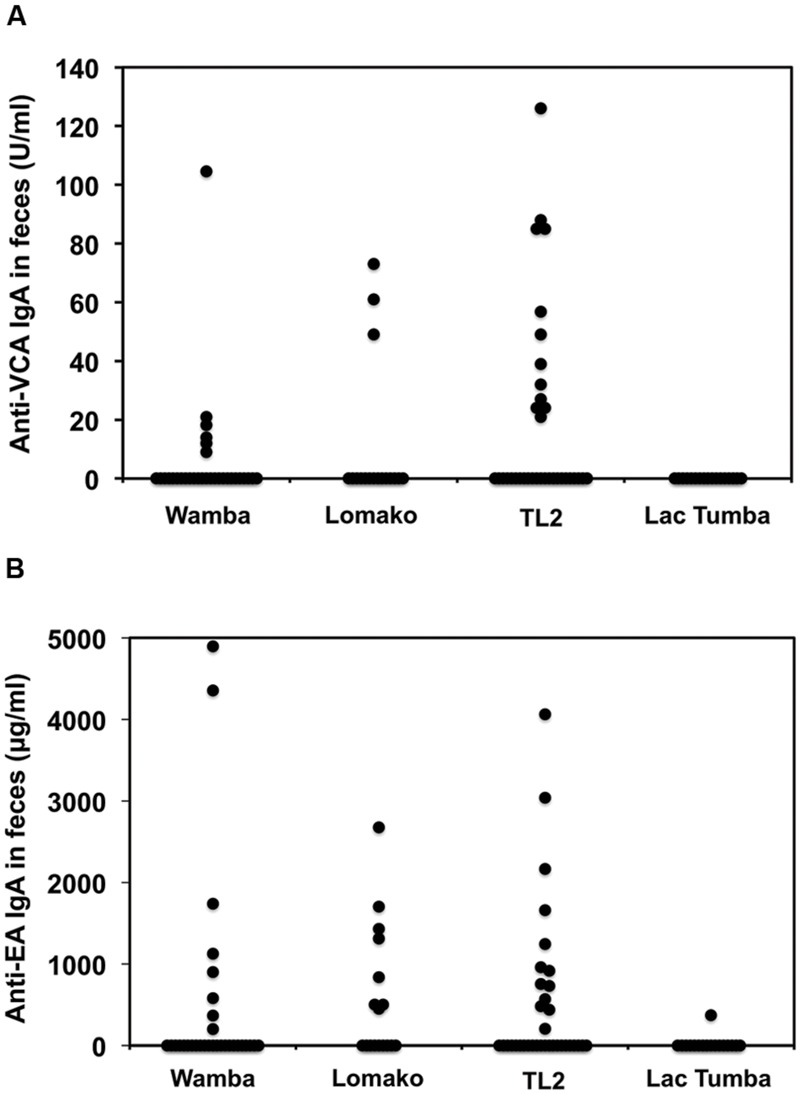
**Comparison among the sampled populations of wild bonobos in terms of the concentration of the anti-VCA and EA IgA antibodies in their feces.** The plotted data indicate the concentration of IgA antibodies against LCV VCA **(A)** and EA **(B)** in the feces of wild bonobos habituated in Wamba, Lomako, TL2, and Lac Tumba, respectively, as measured by ELISA.

Next, we sought to evaluate LCV EA and VCA-specific antibodies in the bonobos’ fecal samples as measured by the ELISA method. As shown in **Table 1**, 21% and 31% of wild bonobos had detectable LCV VCA and EA-specific IgA, respectively. Importantly, the prevalence of LCV antibodies was extensively varied among the sampled populations; 8 of 29 (28%), 8 of 17 (47%), and 13 of 35 (37%) were positive for anti-EA IgA in Wamba, Lomako and TL2, respectively, while only 1 of 17 (0.1%) in Lac Tumba was positive. The results of anti-VCA antibody examination were basically consistent with but less in their titers than those of anti-EA antibody, which was consistent with our finding in captive chimpanzees as shown in **Figures 1** and **2**. The concentration of the anti-VCA and EA antibodies in the feces of bonobos was indicated in **Figure 6**. We did not observe any obvious differences in the IgA concentration among the sampled populations (**Figures 6A,B**).

**Table 1:** Frequency of wild bonobos positive for anti-LCV antibodies in each habitat.

Area	Frequency of individuals positive for VCA antibody	Frequency of individuals positive for EA antibody
Wamba	6/29 (21%)	8/29 (28%)
Lomako	3/17 (19%)	8/17 (47%)
TL2	12/35 (34%)	13/35 (37%)
LacTumba	0/17 (0%)	1/17 (0.1%)

Sum	21/98 (21%)	30/98 (31%)

*VCA and EA indicate viral capsid antigen and early antigen, respectively.*

## Discussion

In the present study, we sought to determine the epidemiological status of LCV in wild bonobos. We first examined whether LCV-specific IgA and IgG antibodies could be induced in the sera from the LCV-infected apes and if so, whether the IgA could be detected in the feces as well as the sera. In parallel, we needed to establish a sensitive detection system for the anti-LCV IgA, which was capable of evaluating fecal samples collected in the different populations of bonobos. We found that IgA against LCV VCA and EA was detectable in both the sera and the feces of seropositive captive chimpanzees (**Figures 1, 2**, and **4**). Considering the genetic proximity of chimpanzees and bonobos, it was reasonable to assume that the LCV-specific IgA in the feces from bonobos would also be detectable in our ELISA system, in the case of the presence of LCV-infected and seropositive bonobos. Fortunately, we were able to observe LCV-specific IgA in the feces of wild bonobos and found that 31% of the individuals tested were positive for the IgA against the EA (**Table 1**), suggesting that the LCV is endemic among bonobos living in DRC. Considering the possibility that the results might contain false positive, i.e., positive for LCV-reactive IgA without LCV infection, we also examined the samples for WB (data not shown). The results of this study generally confirmed the data by ELISA, however, one of 26 samples tested actually showed inconsistent results of ELISA and WB (positive for ELISA while negative for WB). In fact, the positive result of the sample by ELISA was just above borderline, so the discrepancy may be due to lower sensitivity of WB. Taken together, our results in this study indicate the reliability of the ELISA assay for the detection of anti-LCV EA IgA in the feces.

Although, the anti-EA IgG and IgA amounts in the chimpanzee sera were correlated (**Figure 3**), the anti-EA IgA amounts in the sera were not correlated with those in the feces (**Figures 2B,D**). Fecal IgA is derived from mucosal surface of gastrointestinal tract and mainly polymeric with secretory component and J chain (so-called secretory IgA), while serum IgA/IgG is predominantly monomeric. The secretory and serum IgA are produced in different manners and locations, which may be the reason for the incompatibility of the IgA concentration between sera and feces. Alternatively, it is also possible that condition of fecal IgA might be differentially affected depending on the health status of each individual and/or damage of feces when collecting the samples in the wild.

Interestingly, our finding that the prevalence of LCV antibodies were not uniform across bonobos populations and the individuals in Lac Tumba appeared to be almost free of LCV infection while 28–47% of those in other populations were positive (**Table 1**). The results demonstrate that LCV is highly disseminated among wild bonobos while the prevalence is remarkably diverse in their population-dependent manner.

Our findings are significant as they highlight another pressure that threatens the survival of wild bonobos, which are endangered. Infectious diseases, along with poaching and habitat loss present major overlapping and interconnected threats to the survival of African great apes. Naturally occurring zoonotic pathogens, such as Ebola virus, SIV, malaria parasites, as well as anthropozoonotic pathogens causing respiratory diseases are known to contribute to mortality in the great apes (Walsh et al., 2003). Epidemiological information regarding potential life-threatening infectious diseases will be indispensable in the development of conservation strategies for wild bonobos. The protocols that we established in this study for detecting LCV-specific fecal IgA will also be applicable for the surveillance of air-born respiratory viruses infection, by which the presence of the antiviral IgA in the feces may be anticipated. Further epidemiological analyses of the pathogenic agents will provide information as for the risks in the outbreak of infectious diseases including anthropozoonoses, which will help propose evidence-based and optimized policies regarding conservation strategies of wild bonobos, as well as understanding the myriad links between health in the human-wildlife interface.

## Author Contributions

YK, TF, MO, and HA designed research. TY, YE, ES, TK, TM-N, and AS performed experiments. HT, TS, NT, JH, TH, JD, AC, MM, AK, JS, MT, and TM collected or provided animal materials. TY, HT, TF, and HA wrote the paper.

## Conflict of Interest Statement

The authors declare that the research was conducted in the absence of any commercial or financial relationships that could be construed as a potential conflict of interest.

## Footnotes

1 http://www.iucnredlist.org/details/15932/0

2 http://www.bonoboincongo.com/
